# Enhancing Workplace Outcomes Through Digital Behavioral Health Programs: Retrospective Real-World Study

**DOI:** 10.2196/88335

**Published:** 2026-03-04

**Authors:** Inbar Breuer Asher, David L Horwitz, Omar Manejwala, ‪Yifat Fundoiano-Hershcovitz

**Affiliations:** 1 Dario Health Corp Caesarea Israel; 2 DLH Biomedical Consulting Las Vegas, NV United States

**Keywords:** workplace well-being, burnout, evaluation, digital health, behavioral health

## Abstract

**Background:**

Modern workplaces create increasing demands on employees, often leading to stress, burnout, and reduced functional capacity. These challenges contribute to significant functionality losses, with absenteeism and presenteeism posing economic burdens. Mindfulness-based workplace interventions have been shown to improve mental health, yet their effects on specific work-related performance outcomes such as concentration difficulties, mistakes, and procrastination over time are less explored.

**Objective:**

This study aimed to evaluate self-reported workplace function changes for a 10-week period among users of a digital behavioral health program.

**Methods:**

This retrospective analysis used real-world data from users of the Dario Health behavioral health app. Participants were required to complete at least 2 workplace functionality assessments, including one at week 1 as a baseline. The assessment comprised 3 items measuring concentration difficulties, mistakes at work, and procrastination, each rated on a 0 (“not at all”) to 3 (“a lot”) scale. Users who initially scored 2 (“often”) or 3 (“a lot”) on each respective measure formed the cohorts for analysis. Longitudinal changes were examined using piecewise linear mixed-effects models. Cut points were selected empirically through sensitivity analyses comparing alternative cut points (weeks 2-7) using Akaike and Bayesian information criteria to identify the best-fitting model for each outcome.

**Results:**

Sensitivity analyses identified optimal cut points at week 6 for concentration difficulties and procrastination and at week 5 for mistakes at work. Among users reporting baseline concentration difficulties (n=1254), significant reductions were observed during weeks 1 to 6 (B=−0.02; *P*<.001), with larger reductions during weeks 6 to 10 (B=−0.08; *P*<.001). For mistakes at work (n=167), a significant decrease occurred during weeks 1 to 5 (B=−0.05; *P*=.02), followed by a more pronounced reduction during weeks 5 to 10 (B=−0.22; *P*<.001). Among users reporting baseline procrastination (n=1004), significant improvements were observed during weeks 1 to 6 (B=−0.02; *P*<.001), with greater reductions during weeks 6 to 10 (B=−0.08; *P*<.001).

**Conclusions:**

Use of the digital behavioral health platform was associated with improvements in workplace function, particularly among users with sustained use beyond 4 weeks. The observed outcomes suggest that continued participation in such digital behavioral health programs may be linked to favorable changes in mental health and cognitive functioning over time and may support employee well-being. Long-term engagement appears essential to maximize benefits, warranting further research into sustained impacts and optimization strategies for workplace productivity.

## Introduction

In today’s fast-paced work environment, employees are facing escalating demands that can negatively impact their well-being. The increasing demands in the workplace can have adverse effects, leading to work-related stress, exhaustion, burnout, and other health issues [1]. These challenges contribute to significant functional impairments, with absenteeism and presenteeism representing major economic burdens in industrialized countries [2]. Therefore, employers are increasingly implementing health promotion strategies [3], recognizing that employee health and well-being are vital for sustaining functional capacity and ensuring organizational success [4].

Previous studies have shown that mindfulness-based workplace interventions have demonstrated significant benefits in mental health outcomes [5,6]. These programs offer employees accessible and flexible ways to manage stress through mindfulness practices, such as meditation and deep breathing exercises. By integrating these practices into daily routines, employees can develop greater self-awareness and emotional regulation, which help them navigate workplace challenges more effectively. Mindfulness-based interventions have been linked to reductions in symptoms of anxiety, depression, and burnout [7], while improving cognitive functions like attention, memory, and decision-making [8]. As employees feel more supported in managing their mental health, they are better equipped to engage in their work, ultimately benefiting both the individual and the organization.

Workplace mental health has become a critical priority for employers due to its strong association with productivity, absenteeism, presenteeism, and overall organizational performance. While digital behavioral health programs have demonstrated effectiveness in reducing symptoms of depression and anxiety, there remains a limited body of real-world evidence examining their association with functional workplace outcomes, such as concentration, task completion, and error reduction. Digital health measures provide a unique opportunity to evaluate real-world outcomes in workplace function over time and at scale. The objective of this study was therefore to assess changes in user-reported workplace function outcomes over a 10-week period among individuals using the platform in routine practice.

## Methods

### Platform

The Dario Health behavioral health app (DarioHealth Corp) is a modular tool delivering emotion-focused support designed to address a range of mental health conditions. Members generally have access to the Dario Health behavioral health platform as part of their employee or health plan benefits. A clinically based screening tool assesses each person’s needs and guides users to the most efficient support. The app provides several cognitive behavioral therapy programs, including depression, anxiety, anger, stress, and substance use, and can be used as a self-guided intervention or with the help of a certified coach. The structure of each program includes conceptual videos, textual skills, breathing exercises, and progress monitoring tools. These features are designed to address common drivers of workplace impairment, including stress-related attentional difficulties, executive function challenges, emotional dysregulation, and avoidance behaviors that contribute to procrastination. Through repeated skill practice, self-monitoring, and adaptive content delivery, users are supported in building sustainable cognitive and behavioral strategies that may improve concentration, reduce errors, and enhance task completion over time. This study focuses on tracking cohorts of users with work functionality scores measured by their responses to the questionnaire.

### Measures

Workplace functionality levels were tracked for 10 weeks using a 3-item assessment designed to evaluate user-reported issues related to concentration difficulties, mistakes at work, and procrastination (Table 1). The assessment is scored from 0 (not at all) to 3 (a lot).

Internal consistency reliability of the workplace function measure was assessed using Cronbach α. Cronbach α was calculated at baseline assessment, yielding a coefficient of 0.70, indicating acceptable internal consistency. In addition, internal consistency was examined across repeated assessment waves with sufficient sample sizes to assess the stability of the measure over time.

**Table 1:** The components of the assessments and their corresponding answers.

Title	Questionnaire	Scoring
Concentration difficulties	I have not been able to concentrate	0=“not at all” 1=“sometimes” 2=“often” 3=“a lot”
Mistakes at work	I have made avoidable or costly mistakes at work	0=“not at all” 1=“sometimes” 2=“often” 3=“a lot”
Procrastination	I have procrastinated on important work tasks because I just couldn’t get started	0=“not at all” 1=“sometimes” 2=“often” 3=“a lot”

### Ethical Considerations

This study was a retrospective, real-world analysis of an existing database collected in the digital platform. All data were fully anonymized and deidentified before extraction for this study. No identifiable personal information was accessed, and no direct interaction with users occurred for the purposes of this research. In accordance with institutional policies and applicable regulations, the Ethical and Independent Review Services [9], a professional review board, issued the institutional review board exemption for this study (21235-01#).

In accordance with institutional policies and applicable regulations, analyses of fully anonymized retrospective datasets did not constitute human subjects research and therefore did not require institutional review board approval or informed consent. All data were processed in compliance with relevant data protection, privacy, and security standards.

### Study Design

This study used a single-arm, real-world retrospective analysis based on data from users of the behavioral health app. Users included in this analysis were required to have completed at least 2 assessments, including one from week 1. Work productivity outcomes were sorted into 3 different item categories.

### Statistical Analysis

Piecewise linear mixed-effects models were used to examine longitudinal changes in workplace functioning outcomes (concentration difficulties, mistakes at work, and procrastination). This approach was selected to allow for different rates of change across distinct periods, which is appropriate when change is not expected to be uniform over the entire follow-up. Piecewise modeling enables estimation of separate linear slopes before and after a change point, providing a flexible and interpretable framework for analyzing longitudinal trajectories [10]. In the present context, this framework enables evaluation of whether changes in workplace functioning differ between the initial period following baseline and a later period, rather than assuming a single linear trajectory over time. For each outcome, separate models were fitted with time (in weeks since baseline) specified as 2 linear segments divided by a cut point. Models included a random intercept for participants to account for within-subject correlation due to repeated measurements.

To empirically determine the most appropriate cut point, sensitivity analyses were conducted by systematically testing alternative cut points ranging from weeks 2 to 7 for each outcome. For each cut point, models with identical fixed and random effects structures were fitted, differing only in the location of the breakpoint. Model fit was evaluated using the Akaike information criterion (AIC) and Bayesian information criterion (BIC), with lower values indicating better fit. The cut point yielding the lowest AIC and BIC was selected as the optimal cut point for the primary analyses.

Fixed-effect estimates were reported as regression coefficients (B) with 95% CIs and corresponding *P* values. Statistical significance was assessed using 2-sided tests with an α level of .05. All statistical analyses were conducted using R software (R Foundation for Statistical Computing) in RStudio (version 2024.09.0; Posit PBC).

## Results

### Sensitivity Analysis of Piecewise Cut Point Selection

Sensitivity analyses were conducted to evaluate the robustness of the piecewise mixed-effects models to the choice of cut point. Alternative cut points from weeks 2 to 7 were systematically tested for each outcome, with model fit compared using AIC and BIC. These analyses identified week 6 as the optimal cut point for concentration difficulties and procrastination and week 5 as the optimal cut point for mistakes at work, based on minimum AIC and BIC values. Models using these cut points provided the best overall fit to the data.

### Concentration Difficulties

**Figure 1:**
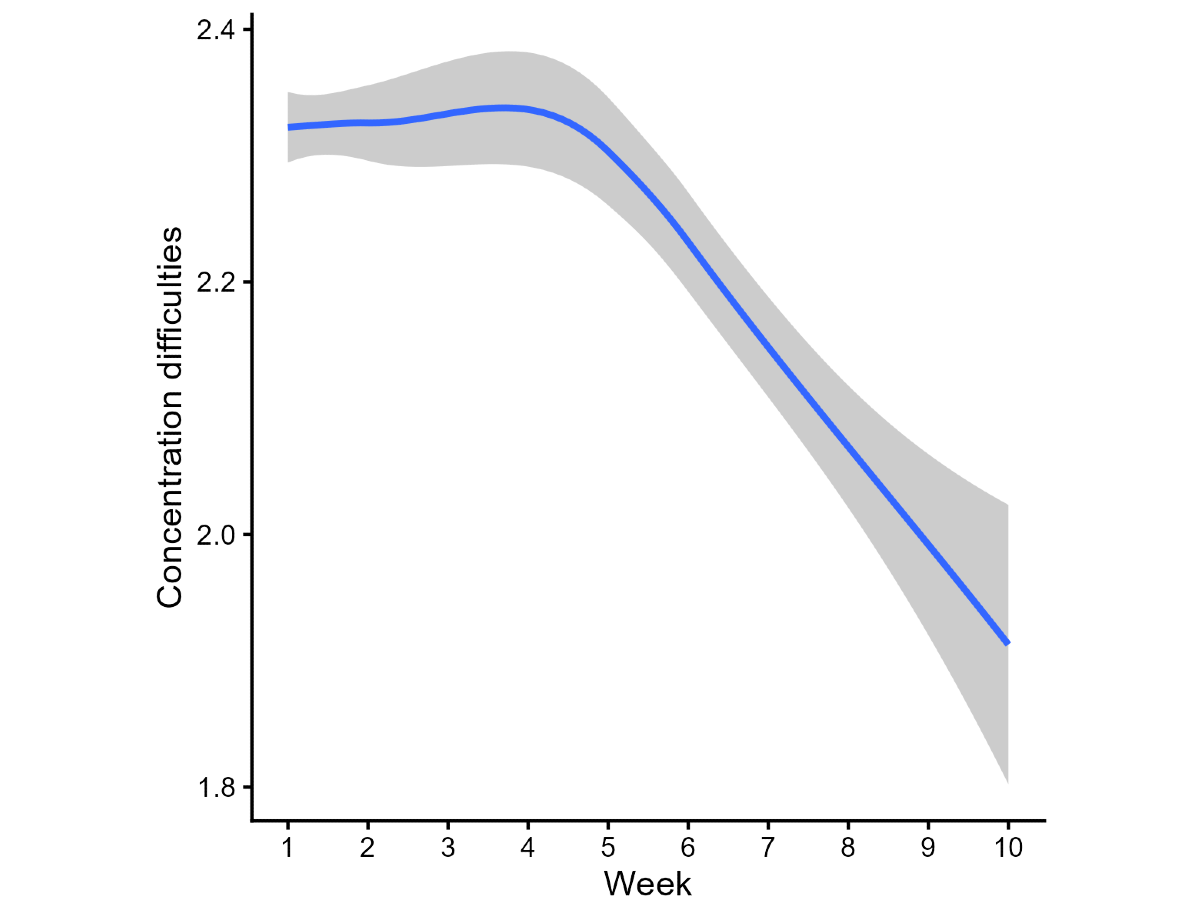
Fluctuation in concentration difficulties over 10 weeks of app use. The blue line represents a smoothing of concentration levels over time. The gray area around the line represents the 95% CI.

The concentration difficulties cohort included 1254 users who reported concentration difficulties (score of 2 [“often”] or 3 [“a lot”]) at baseline. On the basis of model fit criteria, a piecewise mixed-effects model with a cut point at week 6 was selected. The model showed a significant decrease during the first segment (weeks 1-6: B=−0.02, 95% CI −0.02 to −0.01; *P*<.001), followed by a larger and highly significant decrease during the second segment (weeks 6-10: B=−0.08, 95% CI −0.10 to −0.06; *P*<.001; Figure 1). The fitted model predicts a 0.43-point reduction in concentration difficulties scores over 10 weeks.

Mean concentration difficulties scores decreased from 2.33 (SD 0.47) at week 1 to 2.05 (SD 0.72) at week 10, corresponding to an absolute reduction of 0.27 points on the 0 to 3 scale (11.8% decrease).

### Mistakes at Work

**Figure 2:**
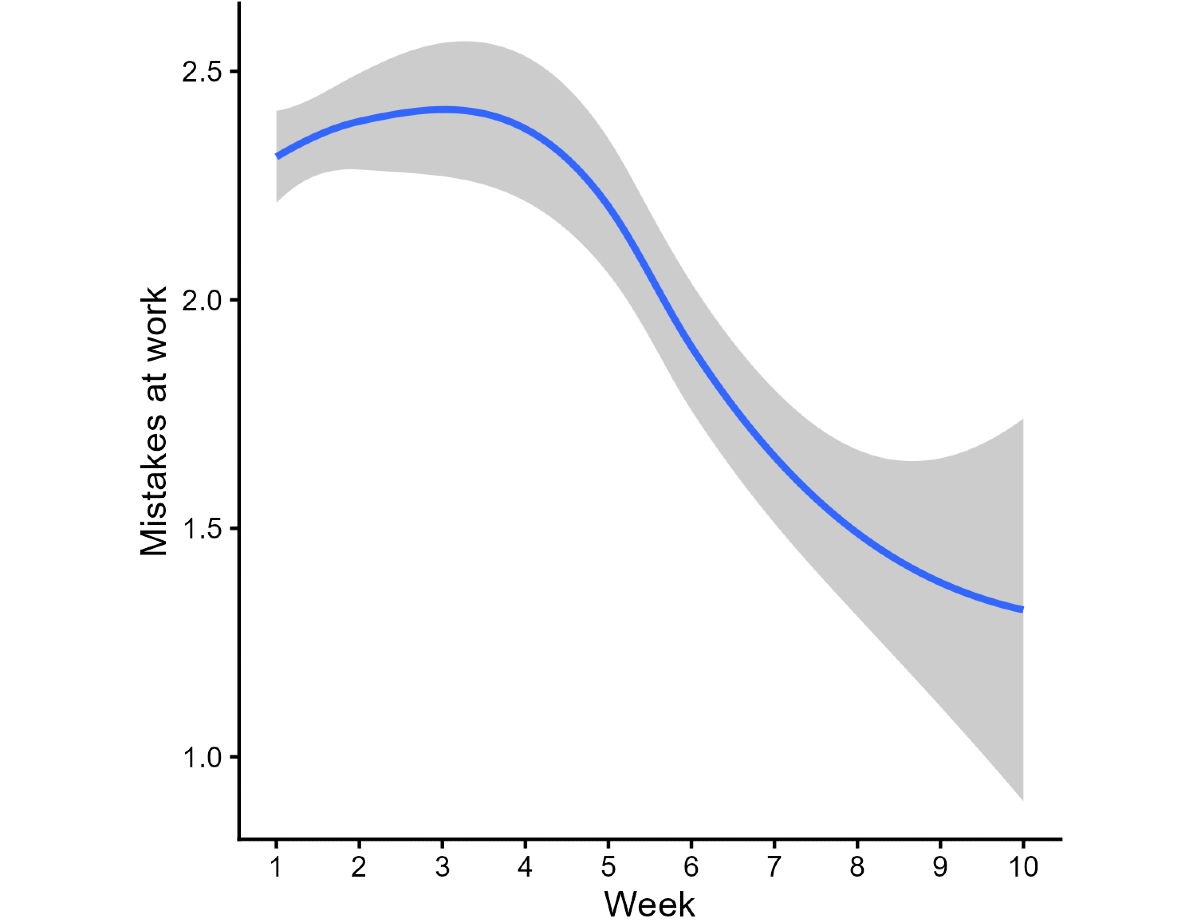
Fluctuation in mistakes at work over 10 weeks of app use. The blue line represents a smoothing of mistakes at work levels over time. The gray area around the line represents the 95% CI.

The mistakes cohort comprised 167 users who reported frequent work-related mistakes (scores of 2 [“often”] or 3 [“a lot”]) at baseline. Model comparison identified week 5 as the optimal cut point. The piecewise mixed-effects model demonstrated a significant decrease during the first segment (weeks 1-5: B=−0.05, 95% CI −0.09 to −0.007; *P*=.02), followed by a more pronounced decrease during the second segment (weeks 5-10: B=−0.22, 95% CI −0.30 to −0.14; *P*<.001; Figure 2). The fitted model predicted a 1.31-point reduction in mistakes at work scores over 10 weeks.

Mean mistake scores decreased from 2.33 (SD 0.47) at week 1 to 1.93 (SD 1.28) at week 10, corresponding to an absolute reduction of 0.40 points on the 0 to 3 scale (17.1% decrease).

### Procrastination

**Figure 3:**
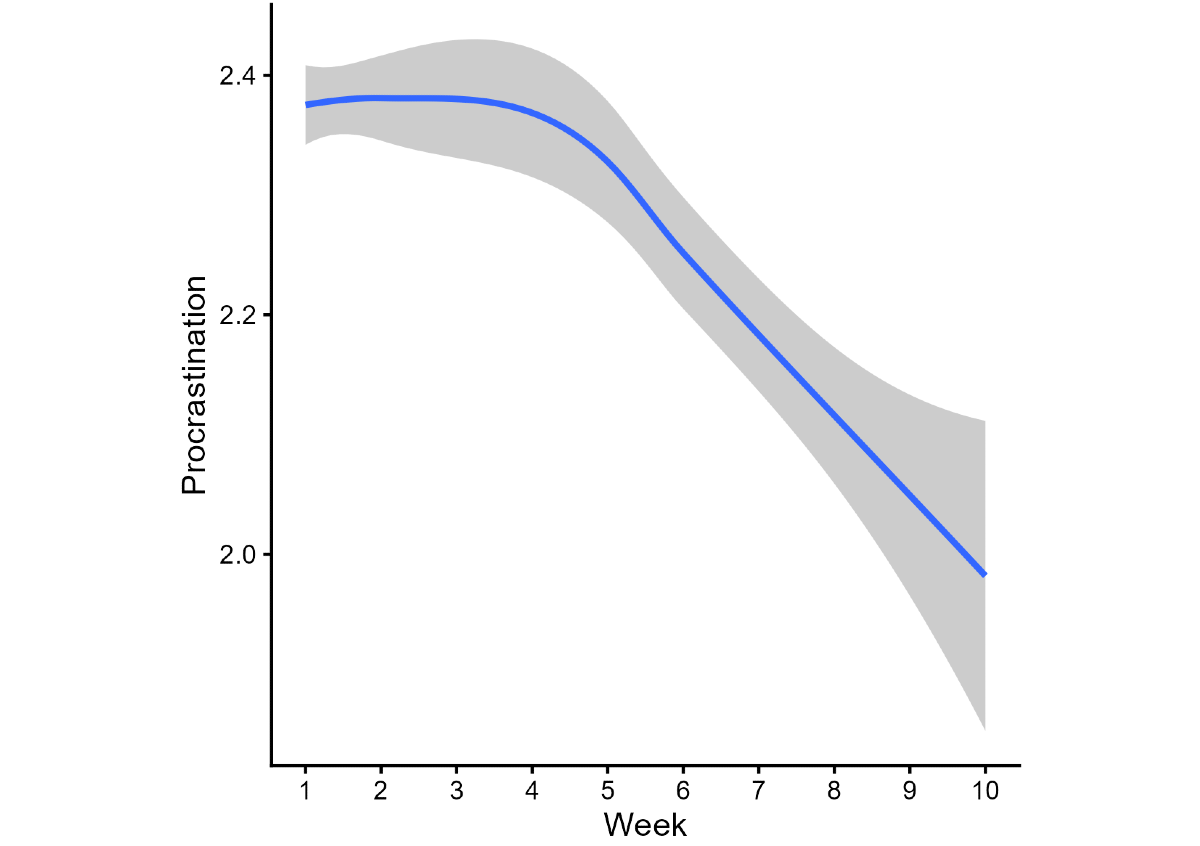
Fluctuation in procrastination over 10 weeks of app use. The blue line represents a smoothing of procrastination levels over time. The gray area around the line represents the 95% CI.

The procrastination cohort included 1004 users who reported frequent procrastination (scores of 2 [“often”] or 3 [“a lot”]) at baseline. A cut point at week 6 provided the best model fit. The piecewise mixed-effects model revealed a significant decrease during the first segment (weeks 1-6: B=−0.02, 95% CI −0.02 to −0.01; *P*<.001), followed by a larger decrease during the second segment (weeks 6-10: B=−0.08, 95% CI −0.11 to −0.06; *P*<.001; Figure 3). The fitted model predicts a 0.44-point reduction in procrastination scores over 10 weeks.

Mean procrastination scores decreased from 2.39 (SD 0.49) at week 1 to 2.01 (SD 0.83) at week 10, corresponding to an absolute reduction of 0.37 points on the 0 to 3 scale (15.7% decrease).

## Discussion

### Principal Findings

The results of this study provide valuable insights into the association between use of the Dario Health digital behavioral health program and improvements in key aspects of workplace function. Achieving significant improvements in workplace functioning is important.

The observed reductions in workplace function outcomes are clinically meaningful when interpreted in the context of established behavioral health symptom scales. At baseline, mean scores across concentration difficulties, mistakes at work, and procrastination ranged from 2.33 to 2.39 on a 0 to 3 frequency scale, corresponding to symptom levels reported as occurring “often” to “a lot.” By week 10, these scores declined to approximately 1.9 to 2.0, reflecting a shift toward lower symptom frequency and functional impairment. Specifically, mean concentration difficulties scores decreased from 2.33 to 2.05 (absolute reduction: 0.27 points; 11.8% decrease), mistakes at work declined from 2.33 to 1.93 (absolute reduction: 0.40 points; 17.1% decrease), and procrastination decreased from 2.39 to 2.01 (absolute reduction: 0.37 points; 15.7% decrease). These magnitudes are comparable to reductions reported in prior digital behavioral health interventions targeting behavioral health conditions, such as depression and anxiety, where symptom improvements of similar relative scale have been associated with meaningful functional recovery and improved daily performance [11-13].

Taken together, these findings suggest that improvements in mental health symptom burden previously demonstrated in our behavioral health programs may translate into parallel gains in cognitive functioning and work-related performance domains, including concentration, task completion, and error reduction.

Following onboarding, users complete an initial assessment of stress, mood, and functional well-being, which informs content recommendations. The platform suggests relevant mindfulness and attention-training activities. Mindfulness content is also available in the app’s content library, allowing members to freely explore and self-select sessions according to their preferences and needs. The mindfulness training itself is delivered through short, structured, evidence-based exercises grounded in mindfulness-based stress reduction and cognitive behavioral therapy principles. These include guided breathing practices, grounding techniques, attention-focusing exercises, and brief meditations designed to support stress regulation, emotional awareness, and cognitive control. Importantly, this study does not evaluate a targeted mindfulness intervention. Rather, it reflects real-world use of a multimodal digital behavioral health platform in which mindfulness is one of several therapeutic modalities available to members. Therefore, engagement with mindfulness practices represents naturalistic, self-directed use within the broader digital care experience.

The delay in the self-observed improvements in workplace function can be attributed to the gradual nature of behavioral interventions. These interventions primarily target mental health outcomes, such as stress reduction and enhanced emotional regulation, which in turn influence cognitive functions such as attention and working memory. The cognitive improvements potentially assist employees in developing better focus, reducing procrastination, and making fewer mistakes. However, the full impact on work-related performance takes time to emerge, as individuals first need to internalize these practices into their routines. This gradual process is consistent with findings in prior research, which showed that improvements in cognitive functions, particularly attention and working memory, were most apparent after completing the full course of mindfulness training [8]. Additionally, another study found that substantial improvements in absenteeism and presenteeism were observed at 8 weeks, compared with the 4-week results [14], suggesting that longer-term engagement with behavioral health interventions leads to more significant reductions in functional impairments.

Workplace productivity is a complex outcome influenced by both mental health and cognitive factors. In this context, the observed improvements in concentration difficulties, decision-making, and overall performance are consistent with the potential benefits of behavioral interventions delivered through a digital health platform [15,16]. These findings also suggest that consistent engagement is essential for employees to report meaningful improvements in their work performance over time.

### Limitations

We noted several limitations in this study. First, as with all retrospective, single-arm, real-world data analyses, groups were not randomly assigned and treatment protocols were not prescribed, creating challenges for drawing causal inferences. Unmeasured confounding factors, such as baseline symptom severity, concurrent mental health treatment, coaching use, job type, work setting, or major life and work-related events, may have influenced changes in workplace function independently of program engagement. Second, participant-level demographic, geographic, and employer-sector characteristics were not available for inclusion, limiting the ability to assess subgroup effects across different workforce populations. In addition, the study relied on self-reported measures of workplace functioning, which are subject to recall bias and social desirability bias. Although self-reported outcomes are highly relevant for subjective experiences such as concentration difficulties and procrastination, they may not fully reflect objective work performance or productivity.

### Conclusions

This retrospective, single-arm, real-world analysis observed improvements in workplace function among users of the Dario Health digital behavioral health program over time. By targeting mental health outcomes first and gradually improving cognitive functions, digital behavioral health programs offer employees a valuable tool for enhancing workplace performance. The findings suggest that sustained engagement with a digital behavioral health platform is associated with favorable outcomes in work-related functioning and mental health outcomes. Future controlled and comparative research should continue to explore the long-term effects of such interventions, further refining strategies for optimizing workplace functionality, productivity, and well-being.

## Abbreviations

AIC: Akaike information criterion
BIC: Bayesian information criterion

## Data Availability

The datasets generated or analyzed during this study are not publicly available due to the company’s privacy policy but are available from the corresponding author on reasonable request, subject to company policies.
